# Grasping follows Weber's law: How to use response variability as a proxy for JND

**DOI:** 10.1167/jov.22.12.13

**Published:** 2022-11-14

**Authors:** Kriti Bhatia, Christian Löwenkamp, Volker H. Franz

**Affiliations:** 1 Experimental Cognitive Science, University of Tübingen, Tübingen, Germany; 2 Department of Psychology, University of Hamburg, Hamburg, Germany

**Keywords:** psychophysics, grasping, Weber's law, manual estimation, perception–action model

## Abstract

Weber's law is a fundamental psychophysical principle. It states that the just noticeable difference (JND) between stimuli increases with stimulus magnitude; consequently, larger stimuli should be estimated with larger variability. However, visually guided grasping seems to violate this expectation: When repeatedly grasping large objects, the variability is similar to that when grasping small objects. Based on this result, it was often concluded that grasping violated Weber's law. This astonishing finding generated a flurry of research, with contradictory results and potentially far-reaching implications for theorizing about the functional architecture of the brain. We show that previous studies ignored nonlinearities in the scaling of the grasping response. These nonlinearities result from, for example, the finger span being limited such that the opening of the fingers reaches a ceiling for large objects. We describe how to mathematically take these nonlinearities into account and apply this approach to our own data, as well as to the data of three influential studies on this topic. In all four datasets, we found that—when appropriately estimated—JNDs increase with object size, as expected by Weber's law. We conclude that grasping obeys Weber's law, as do essentially all sensory dimensions.

## Introduction

“Weber's law is the first and still most widely tested (and confirmed) formal principle in modern psychological science” (Baird & Noma, 1978; Ganel et al., 2008, p. R599) and can be found in almost all sensory dimensions (Teghtsoonian, 1971), including visual size perception. Weber's law states that the just noticeable difference (JND) between two stimuli increases with stimulus magnitude (Baird & Noma, 1978, equation 4.1; Fechner, 1860).

Given the ubiquity of Weber's law it was very astounding when researchers reported that grasping—a central human ability—does not obey Weber's law (Ganel et al., 2008). The main experimental result leading to this claim has been replicated many times, and far-reaching theoretical consequences for the understanding of the functional architecture of the brain were derived (Ayala, Binsted, & Heath, 2018; Christiansen, Christensen, Grünbaum, & Kyllingsbæk, 2014; Freud, Culham, Namdar, & Behrmann, 2019; Ganel et al., 2008; Hadad, Avidan, & Ganel, 2012; Heath, Mulla, Holmes, & Smuskowitz, 2011; Heath, Holmes, Mulla, & Binsted, 2012; Heath, Manzone, Khan, & Davarpanah Jazi, 2017; Heath & Manzone, 2017; Holmes, Mulla, Binsted, & Heath, 2011; Holmes & Heath, 2013; Hosang, Chan, Jazi, & Heath, 2016; Jazi & Heath, 2017; Löwenkamp, Gärtner, Haus, & Franz, 2015; Namdar, Algom, & Ganel, 2018; Ozana, Berman, S., & Ganel, 2018; Ozana & Ganel, 2017; Ozana & Ganel, 2018; Utz, Hesse, Aschenneller, & Schenk, 2015).

We will first describe the rationale that leads to the claim of a violation of Weber's law in grasping. Then, we will show that this rationale does not account for the nonlinear scaling of grasping as a function of physical object size. When this nonlinear scaling is appropriately taken into account, then grasping does obey Weber's law. This is so in our own experiment (specifically designed to test these issues), as well as in reanalyses of three published studies, including the original landmark study by Ganel et al. (2008). Finally, we will discuss consequences for the far-reaching theoretical implications that have been derived from the violation of Weber's law in grasping.

### The initial finding: A violation of Weber's law in grasping

Ganel et al. (2008) were the first to report that Weber's law is violated in grasping. Participants performed three tasks: one grasping task and two perceptual tasks. The grasping task seemed to violate Weber's law but the perceptual tasks seemed to obey Weber's law. In the grasping task, participants grasped objects of different sizes, and Ganel et al. (2008) measured the maximum grip aperture (MGA). This is the maximum opening between the index finger and thumb during grasping and is a function of physical object size: the larger the object, the larger the MGA (Franz, 2003; Hesse & Franz, 2009; Jeannerod, 1984; Smeets & Brenner, 1999). Ganel et al. (2008) then calculated the within-subjects standard deviation of the maximum grip aperture (*SD*_MGA_) as a proxy for the corresponding JND and found that *SD*_MGA_ does not increase with object size. From this they concluded that Weber's law is violated in grasping.

In the perceptual tasks, participants either adjusted a comparison line on a monitor to match the size of visually presented objects (perceptual adjustment) or indicated the size of these objects with the span between the index finger and thumb (manual estimation).^1^ Again, Ganel et al. (2008) calculated the within-subjects standard deviations of each of these responses (*SD*_Response_) as a proxy for the corresponding JND and found for both perceptual tasks that *SD*_Response_ did increase with object size. From this, they concluded that Weber's law holds for perceptual tasks—in accordance with the well-known ubiquity of Weber's law in most sensory dimensions (Teghtsoonian, 1971).

In short, Ganel et al. (2008) used for each task the within-subjects standard deviations of the response (*SD*_Response_) as a proxy for the corresponding JND, as did subsequent studies on Weber's law in grasping. However, we will show that this approach is only valid if there is a perfectly linear relationship between stimulus and response. Any small nonlinearity will make this approach problematic and can lead to erroneous conclusions. For ease of exposition, we will focus on grasping, where the typically measured response is MGA, such that the within-subjects standard deviation of the response is *SD*_MGA_, but all our arguments apply equally well to other tasks and responses. For the sake of generality, we use the term *SD*_Response_ to subsume multiple possible responses but refer to *SD*_MGA_ in concrete cases of grasping. Before describing why it is problematic to use *SD*_MGA_ as a proxy for JND, we first need to describe a more general problem.

### A subtle pitfall: The erroneous equalization of stimulus and response

Studies on grasping often use MGA because of its strong dependence on physical object size: the larger the object, the larger the MGA. This allows for relatively straightforward inferences about which object size was used by the motor system to prepare a movement. Nevertheless, some care needs to be applied when making such inferences. This is so, because MGA is not *identical* to the object size. For example, it is well known that the MGA is always larger than the to-be-grasped object, such that there is a safety margin (cf. Uccelli, Pisu, & Bruno, 2021) that prevents the fingers from colliding with the object (for a laborious measurement of this response function, see Figure 6a of Hesse & Franz, 2009; for a comprehensive review, see Figure 6a of Smeets & Brenner, 1999).

Here is a simple example that demonstrates the potential pitfalls. Consider that researchers presented multiple objects of different sizes (e.g., blocks of different lengths) to a participant and measured the response function: MGA as a function of object size. The response function would show the customary safety margin: MGA is always larger than the target object. Now, assume the participant grasped one of the objects, but the researchers did not know which object. All that the researchers knew was that the grasp was performed with an MGA of 70 mm. What should the researchers conclude about the object size for which the motor system prepared this grasp?

Given the researchers’ knowledge about the response function and the safety margin, it would be an obvious mistake to believe the motor system had prepared for a 70-mm object. Instead, they have to correct for the safety margin. This can easily be done by assessing the response function to see which object size typically corresponds to an MGA of 70 mm. A typical value for the safety margin could be 40 mm, such that the researchers would arrive at the correct conclusion that the motor system had prepared for a 30-mm object.

Had researchers, however, inferred an object size of 70 mm from an MGA of 70 mm, then they would be *erroneously equating the response with the stimulus*, because they would be confusing the response (here, MGA) with the stimulus (here, physical object size that prompted the motor system to prepare the grasp) by implicitly assuming that those two were identical. Instead, what the researchers should do is to *invert* the response function. Whereas they first measured MGA as a function of object size, now they needed to calculate object size as a function of MGA in order to *find the stimulus that elicited the response*. We will see that a similar problem exists when *SD*_MGA_ is used as proxy for JND.

### Why was *SD*_MGA_ used as proxy for JND?

When Ganel et al. (2008) wanted to assess whether grasping obeys Weber's law, they had a problem: It is not clear how to assess Weber's law in grasping. Weber's law requires an experiment where a participant *compares* two objects of different sizes and decides whether they are of equal or different sizes. The JND is then the difference in physical sizes between the two objects at which the participant responds 50% of the time “different” and 50% “equal.” Weber's law states that the JND is (roughly) proportional to the absolute size of the object. However, such a comparison is not possible in grasping and even less so in “natural grasping” (target-oriented grasping; see Goodale, Jakobson, & Keillor, 1994) as is prescribed by the perception–action model if one wants to measure the dorsal stream (see General discussion). These problems arise because grasping is typically targeted at a single object and does not easily allow for a comparison of two objects.

The solution of Ganel et al. (2008) was to assess the within-participants standard deviation of the response (*SD*_MGA_ for grasping) as a proxy for JND. Following their lead, subsequent studies also based their investigations of Weber's law in grasping on *SD*_MGA_ or on similar measures (see General discussion). However, we will show that this choice again constitutes an erroneous equalization of response and stimulus because *SD*_MGA_ is related to the variability in the *response*, whereas JND is related to the variability of the *stimulus* (it gives us the amount we would have to change the physical object until this change is detected).

### A more principled approach to arrive at a proxy for JND

**Figure 1.**
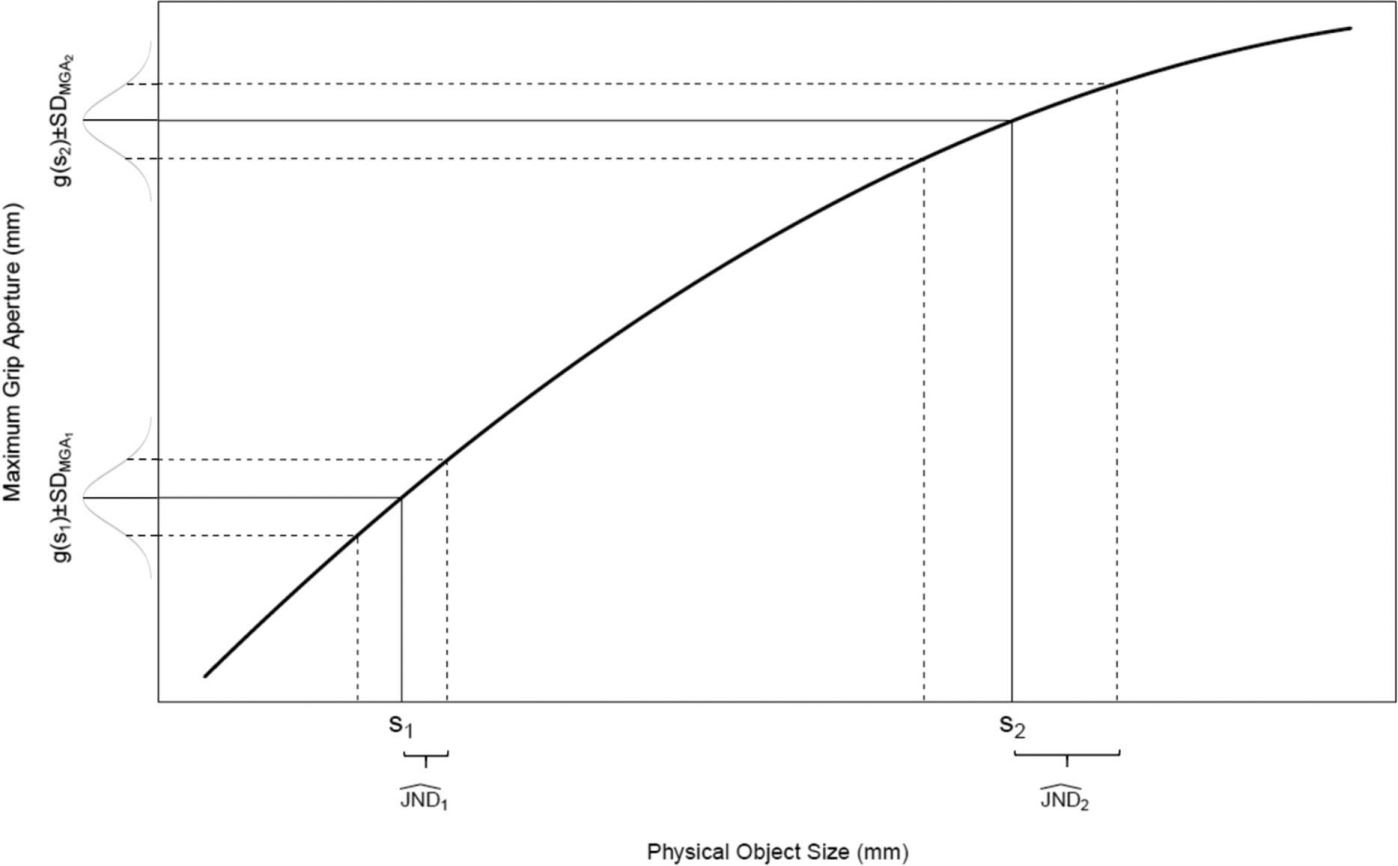
Illustration of the apparent violation of Weber's law in grasping due to the nonlinear response function *g*(*s*). First, suppose we already knew that Weber's law holds in grasping, such that uncertainty about object size is smaller for small objects than for large objects (compare ${\hat{JND}}_{1}$ with ${\hat{JND}}_{2}$, respectively). This larger uncertainty is, however, not necessarily reflected in the variability of the response because the response function in grasping becomes shallower for large objects (compare *SD*_MGA_1__ and *SD*_MGA_2__, respectively). This illustrates how the nonlinear response function can mask an underlying adherence to Weber's law in grasping. Now, consider what needs to be done if a researcher only knows *SD*_MGA_ and the response function but not $\hat{JND}$. The researcher would need to invert the response function and map *SD*_MGA_ back to the corresponding uncertainty at the level of object size. Only then would the researcher arrive at the correct estimates for $\hat{JND}$. For mathematical details, see Appendix A.

To see what a more appropriate solution looks like, consider the following situation: A participant is presented with a stimulus of size *s*_1_ and responds with a certain MGA_1_ and *SD*_MGA_1__ (Figure 1). Now, we increase the stimulus until this change can be detected in the response. For this detection, we have to set a certain threshold, but this is not critical and standard practice in signal detection models. Therefore, let us set the threshold to ±1 *SD*_MGA_1__, such that we consider the physical size change as being detected when it creates a change in MGA that corresponds to 1 *SD*_MGA_1__ (our results would not change had we used a factor other than 1 here). Now, all we have to do is determine how much we have to change the stimulus to create this 1-*SD*_MGA_1__ change. To determine this, we have to invert the response function and map *SD*_MGA_1__ back to the stimuli. Let us call the change in stimulus size that is needed to elicit a 1-*SD*_MGA_1__ change the ${\hat{JND}}_{1}$ (for “estimated JND”). This ${\hat{JND}}_{1}$ tells us how much we would need to change the stimulus *s*_1_ to detect a 1-*SD*_MGA_1__ change in the response. Appendix A gives the corresponding math: All one has to do is to calculate at every object size the local slope (the first derivative) of the response function that relates object size to MGA and then to divide the *SD*_MGA_1__ by this local slope to obtain ${\hat{JND}}_{1}$ (the math is similar to the famous error propagation formula in statistics). We then can use ${\hat{JND}}_{1}$ as a proxy for the JND for this stimulus.

However, Ganel et al. (2008) used *SD*_MGA_ directly as a proxy for JND. Thereby, they equated the MGA with the physical stimulus size, erroneously equating response and stimulus. In a nutshell: Their intuition to use an *SD* as a proxy for JND is acceptable, but they used the wrong *SD*—at the level of the response, rather than the stimulus.

Still, the use of *SD*_MGA_ instead of $\hat{JND}$ would not have dramatic consequences if the response function that relates object size to MGA were perfectly linear. In that case, all local slopes are equal (i.e., for each object size the response function has the same slope) and the transformation from *SD*_MGA_ to $\hat{JND}$ is always by the same constant factor (because we always divide by the same slope). Therefore, *SD*_MGA_ could still be used as a proxy for JND when one wanted to assess Weber's law. However, in grasping the response function is not linear. This slight nonlinearity has relatively large effects when trying to use *SD*_MGA_ as a proxy for JND instead of $\hat{JND}$.

To understand the effects of the slightly bent response function, consider the second stimulus with physical size *s*_2_ in Figure 1. This stimulus has exactly the same *SD*_MGA_ as the stimulus with size *s*_1_, but, because the response function is slightly bent, we have to change the physical size of *s*_2_ much more than that of *s*_1_ to achieve the same effect on the MGA. Although *SD*_MGA_2__ and *SD*_MGA_1__ are identical (which would be interpreted by Ganel et al., 2008 as a violation of Weber's law), ${\hat{JND}}_{2}$ is much larger than ${\hat{JND}}_{1}$—just as expected by Weber's law!

Of course, the response function could be bent even more. In this case, it is easily possible that *SD*_MGA_ is even smaller for large than for small stimuli (Bruno, Uccelli, Viviani, & Sperati, 2016; Löwenkamp et al., 2015; Utz et al., 2015), and nevertheless Weber's law could still hold (i.e., $\hat{JND}$ could still increase as predicted by Weber's law). All this can only be tested and decided when the appropriate proxy for JND is used.

### Overview of our study

We have shown that nonlinear effects in the scaling of the grasping response can erroneously mask Weber's law when *SD*_MGA_ is used as a proxy for JND. To avoid this pitfall, researchers need to first calculate $\hat{JND}$ and use this as a proxy for JND. Only then does it make sense to draw inferences about Weber's law. In the following, we will apply this approach to grasping and manual estimation using four different datasets: Experiment 1 consists of newly collected data using a design that was specifically optimized for this purpose. Then, we present three reanalyses of two previously published studies (Heath et al., 2011; Löwenkamp et al., 2015) and the pioneer study on this subject (Ganel et al., 2008).

We chose the studies for reanalysis with the following logic: Löwenkamp et al. (2015) was chosen because we had the full data available, and all methodological details were known. Ganel et al. (2008) was reanalyzed because it was the first landmark study on this topic, and we tried to replicate their findings. Next, we looked for highly cited studies investigating grasping and Weber's law that had at least 10 participants and 20 trials per object size and where the data was either available or given in tables. The most cited study that fulfilled these criteria was Heath et al. (2011), and this research group has contributed a lot to grasping and Weber's law; therefore, it is representative of studies in the field to reanalyze their work.

We will show that when our analysis is applied grasping is consistent with Weber's law. That is, $\hat{JND}$ increases with object size in a linear fashion, and the corresponding slope is in the range that can be expected from the literature for size estimation (i.e., the Weber constant *k* is between 0.02 and 0.06, as we would expect from classic studies) (cf. McKee & Welch, 1992; Teghtsoonian, 1971).

## Experiment 1: Weber's law in grasping and manual estimation

First, we conducted our experiment with a grasping and a manual estimation task. The design was optimized to investigate Weber's law: (a) we minimized biomechanical constraints by using functionally “graspable” object sizes between 20 and 50 mm (Ayala et al., 2018; Heath & Manzone, 2017; Heath et al., 2017); (b) each object was repeated 50 times (instead of the usual ≤20 repetitions in such experiments; cf. Appendix D) to improve the parameter estimates; and (c) we used a relatively large sample size of *N* = 20 participants. We calculated $\hat{JND}$ as described above and in Appendix A. In a nutshell, at each object size and for each participant, we divided the within-subjects standard deviation of the responses (*SD*_Response_) by the local slope of the response function (Figure 1), resulting in $\hat{JND}$. Weber's law holds when $\hat{JND}$ increases linearly with object size.

### Methods

#### Participants

Twenty participants (14 females, 6 males; age range, 19–38 years) took part in the grasping task. Twenty new participants (17 females, 3 males; age range, 18–36 years) took part in the manual estimation task. Participants were either undergraduate students who received course credits or paid volunteers, were native German speakers, were self-declared right-handed dominant, and had normal or corrected-to-normal vision.

#### Ethics statement

Written informed consent was obtained from all participants. The study was conducted in accordance with the tenets of the Declaration of Helsinki and in keeping with the ethical guidelines of the Professional Association of German Psychologists (2005, C.III) and the German Psychological Society. This study was conducted within the International Graduate Research Group “Cross-Modal Interaction in Natural and Artificial Cognitive Systems,” which was reviewed and approved by the German Research Foundation (project no. IGK-1247).

#### Apparatus and procedure

**Figure 2.**
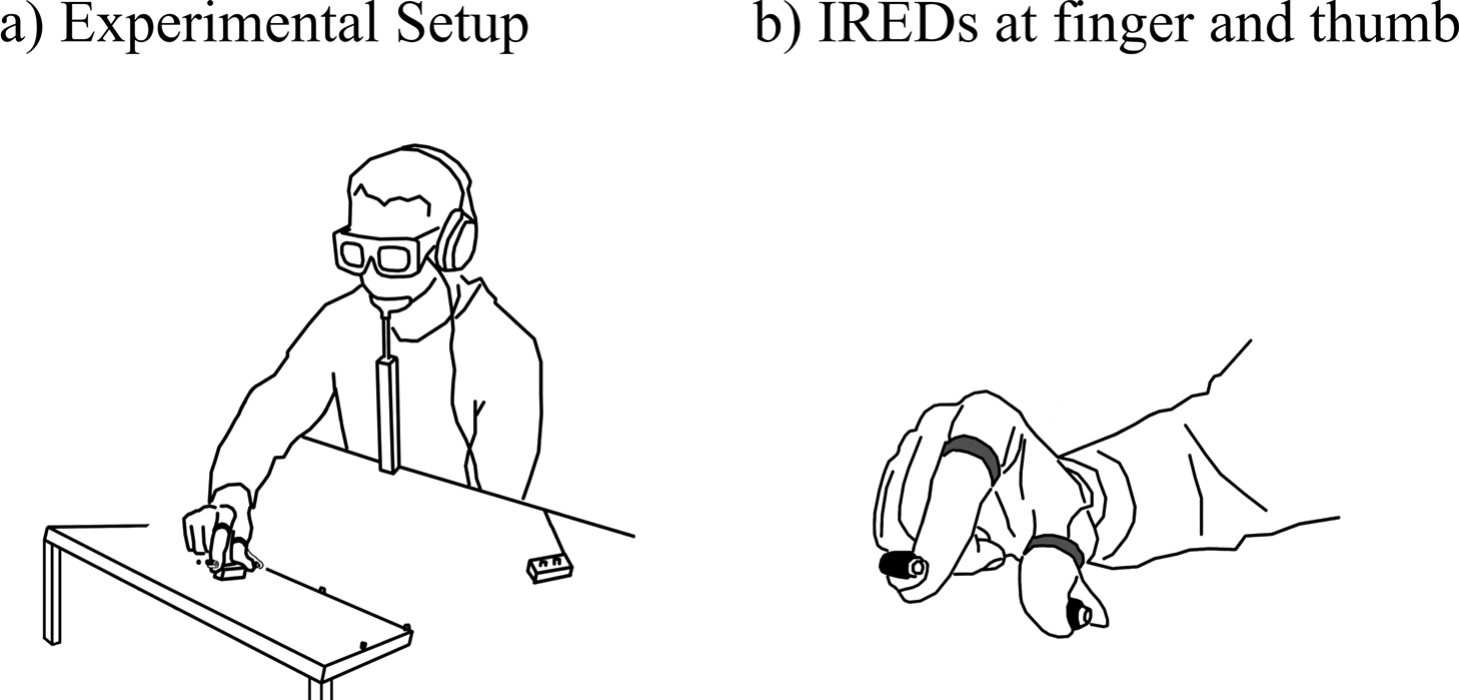
(a) Setup of Experiment 1 for grasping and manual estimation. (b) Infrared light-emitting diodes (IREDs) were fixed to the index finger and thumb to record the trajectories of the movements.

The experimental setup is depicted in Figure 2a. Each participant sat at a table with his or her head positioned in a chin rest. To control the timing of visual presentation, the participants wore liquid-crystal shutter goggles (PLATO; Translucent Technologies, Inc., Toronto, ON, Canada). To present the acoustic start signal and shield from possible sounds of stimulus placement, participants wore headphones with isolation against ambience attenuation of 35 dB (DT 770 M, 80 ohm; beyerdynamic, Heilbronn, Germany). Target objects were plastic blocks that were 20, 30, 40, and 50 mm in length and 15 mm in width and depth. They were loosely attached at a 40° sloped platform.

At the beginning of each trial, participants placed their right index finger and thumb pinched together at a start position on the sloped platform 3 cm to the right of the target object. We used a short distance between start position and target object to reduce the amount of motor noise in the transport component of the grasping response. When the experimenter pressed a button, the shutter goggles became transparent and enabled full vision of the target object lying on the sloped platform. Participants were prompted to respond by a 1000-Hz tone after a fixed time interval of 960 ms plus a random time interval drawn from an exponential distribution with a mean of 240 ms (see also Löwenkamp et al., 2015).

In the grasping task, participants grasped the object with the index finger and thumb of the right hand. Movement onset caused the shutter goggles to close, preventing sight of the object during grasping (open-loop grasping). After lifting the object and putting it on the desk in front of the sloped platform, participants returned their finger and thumb to the start position. The goggles remained closed until the experimenter set up the next object and started the following trial.

Here is a short justification of a few design choices used in our experiment: Open-loop grasping was chosen because it allows assessment of visuomotor responses based solely on initial visual information, independent of online visual feedback (Haffenden & Goodale, 1998; Post & Welch, 1996). Furthermore, it is typically not problematic or unusual for participants to perform open-loop grasping, and it has been shown that eye movements “often moved on to the next object in the sequence before completion of the preceding action” (Land & Hayhoe, 2001, p. 3559). A between-subjects design was used, because it is common practice in this field and was also used by the pioneer study by Ganel et al. (2008) and the other study we reanalyzed (Heath et al., 2011). We also focused on JNDs at the time of MGA, and not 100% of movement time, because MGA is not contaminated by physical contact with the target object, which biases the response heavily to the true physical size. We employed a “natural” grasping task that involved grasping a physically present, three-dimensional (3D) object in a real set-up with haptic feedback provided on contact with the object. When grasping an object in a virtual environment or if the object is two dimensional (2D) or not physically present (pantomimed or simulated grasping) or when no feedback is provided on grasping the object, it is assumed by the perception–action model that “stored perceptual information” is used, due to which one would a priori assume Weber's law in such kinds of “unnatural” grasping (Goodale et al., 1994).

In the manual estimation task, participants moved their right hand approximately 5 cm to the right of the start position and performed manual estimation by indicating the visual size of the object with the span between index finger and thumb, as accurately and spontaneously as possible. Movement onset caused the shutter goggles to close, preventing sight of the object during manual estimation (open-loop manual estimation) (Haffenden & Goodale, 1998). Participants indicated when they were showing the size of the target object by pressing a button with the left hand. If the button press did not occur within 2.5 seconds after the start tone or if the movement velocity between the index finger and thumb at the time of the button press was larger than 30 mm/s (see Franz, 2003), then the trial was considered invalid and was repeated at a random later time. After estimating the target object (without returning to the start position) participants grasped the object. This was performed to provide a similar amount of haptic feedback in manual estimation as in grasping and is a standard procedure (Ganel et al., 2008; Haffenden & Goodale, 1998; Heath et al., 2011; Holmes et al., 2011; Löwenkamp et al., 2015). After lifting the object and putting it on the desk in front of the sloped platform, participants returned their fingers to the start position. The goggles remained closed until the experimenter set up the next object and started the following trial. In both tasks, the four target objects were presented randomly, and each of the target objects was repeated five times during practice trials (i.e., 20 trials) and 50 times during experimental trials (i.e., 200 trials).

An Optotrak Certus (Northern Digital, Inc., Waterloo, ON, Canada) with a sampling rate of 200 Hz was used to record the trajectories of the infrared light-emitting diodes (IREDs). Three IREDs were placed on the platform for spatial reference. Two IREDs were fixed with adhesive putty (UHU-Patafix; UHU GmbH, Bühl, Germany) on the fingernails of the index finger and thumb (Figure 2b). Control of stimulus presentation and data recording was obtained with the Psychophysics Toolbox (Brainard, 1997) and the Optotrak Toolbox (http://www.ecogsci.cs.uni-tuebingen.de/OptotrakToolbox) within MATLAB (MathWorks, Natick, MA).

#### Data analysis

Movement onset was determined when at least one IRED crossed a sphere with a radius of 30 mm around the start position and movement velocity in at least one IRED exceeded 25 mm/s. Movement offset was determined as the time of the first contact with the object (either thumb or index finger). For this purpose, a mirror foil was mounted at each target object reflecting the infrared signal of an IRED mounted 2 cm to the left of the target object. This mirror image was tracked by the Optotrak and allowed recording of the subtlest movements of the object (Franz, Scharnowski, & Gegenfurtner, 2005). The first contact with the object (movement offset) was determined as the time when the velocity of the mirror image of the IRED exceeded 6 mm/s. MGA was defined as the peak distance between the IREDs of the index finger and thumb between movement onset and offset. The response in manual estimation (ME) was calculated as the distance between the finger and thumb of the participant's right hand at the time of the button press. A trial was considered invalid and repeated randomly later in the experiment if movement onset occurred before the start signal or if an IRED was occluded. In grasping, 23 trials of the 4000 trials were excluded because MGA equaled the aperture at the time of movement offset, indicating that the MGA occurred at the time of or after the contact with the object. This was done to prevent any corrections of the MGA using feedback given by physical contact with the object. Nonetheless, we also analyzed the data including these trials and obtained essentially identical results. One trial was excluded because the MGA was not recorded. Practice trials were not included in the analyses (we also analyzed the data including practice trials, which produced essentially the same results).

Mean, standard deviation, and skewness (type 2) (Joanes & Gill, 1998) of the response were calculated for each object size and participant. Quadratic regressions (*g*(*s*) = *a* + *bs* + *cs*^2^) were fitted for each participant (Appendix A). To allow for a meaningful interpretation of the linear term *b* of the quadratic regression, the predictor (i.e., size) was centered on its mean, such that the linear term *b* of the quadratic regression describes the slope at the mean object size (i.e., 35 mm) and equals the slope *b* of a simple linear regression. $\hat{JND}$ was calculated by dividing the within-subjects standard deviation of the response by the local slope of the participant's individual quadratic regression function at each size for each participant (Appendix A). Linear regressions relating $\hat{JND}$ to object size were then fitted for each participant in order to assess Weber's law. The linear regression allowed for a non-zero intercept. Strictly speaking, Weber's law does not include an intercept. However, it is known that the generalized form of Weber's law (which includes an intercept) is a better descriptor of behavior, and it is standard practice to model Weber's law with a non-zero intercept (Baird & Noma, 1978; Brown, Galanter, Hess, & Mandler, 1962; Miller, 1947). We discuss this issue further below.

Analyses were conducted using R (R Core Team, 2022), ggplot2 (Wickham, 2011), and MATLAB. For all analyses, a significance level of α = 0.05 was applied, and *p* values of 0.001 or less are depicted as *p* < 0.001. Between-participant means and corresponding standard errors are depicted as mean ± 1 *SEM.* We also report 95% confidence intervals (CIs) for the slopes of the linear regression of *SD*s and $\hat{JND}$s on object size (for the studies where we have the full data).

### Results and discussion

#### Grasping

**Figure 3.**
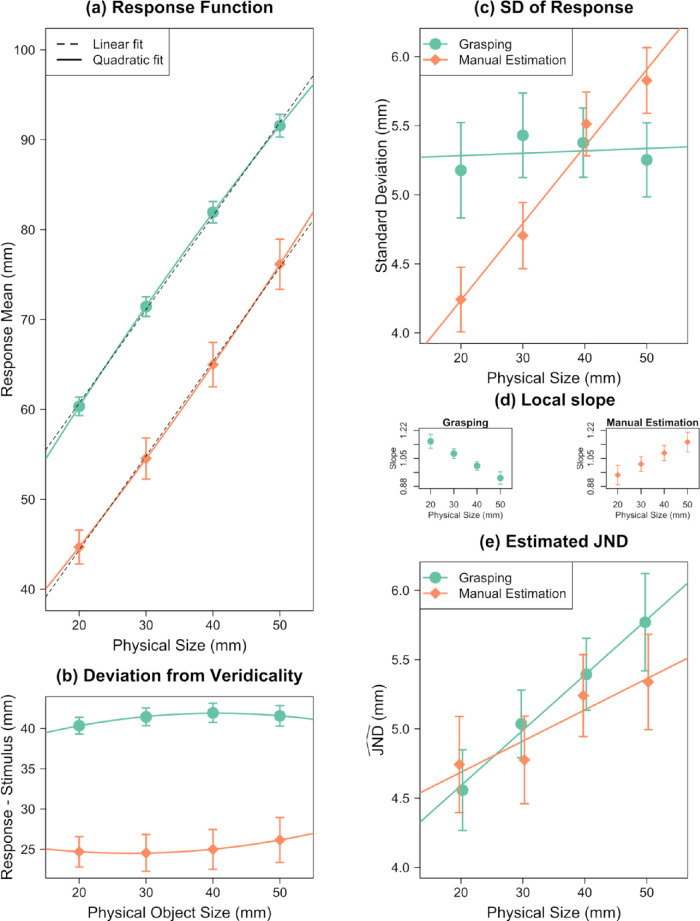
Results of Experiment 1 for grasping and manual estimation. (a) Mean responses as a function of object size. Quadratic regressions are shown as solid color curves; linear regressions as black dashed lines. (b) Difference between response (and quadratic fit) and physical stimulus size. The nonlinearity in the responses can be seen in the curvature of these differences. (c) *SD*_Response_ as a function of object size. (d) The local slope at every object size in the grasping and manual estimation. This is the value that the *SD* is divided by to calculate $\hat{JND}.$ (e) $\hat{JND}$ as a function of object size. Error bars depict ±1 *SEM* (between subjects).

Results are summarized in Figure 3, and regression coefficients are listed in Appendix C. The quadratic regressions revealed a curvilinear relationship between MGA and object size (Figure 3a). Figure 3b shows the difference between response and physical object size. The linear coefficient was *b*_MGA_ = 1.04 ± 0.013, and the quadratic coefficient was *c*_MGA_ = −0.0037 ± 0.0005 mm^−1^. The residuals of the linear and quadratic fits are depicted in Appendix B. The residuals of the linear fit indicate a systematic relationship, which disappears in the residuals of the quadratic fit. The sign of the quadratic coefficient was negative, indicating a concave relationship between MGA and object size: The responsiveness of MGA decreased with increasing object size.

*SD*_MGA_ did not scale with object size, *b* = 0.002 ± 0.010, *t*(19) = 0.18, *p* = 0.859, 95% CI, −0.018 to 0.022 (Figure 3c). Thus, we replicated the finding of previous studies that the uncertainty of the response in grasping does not increase with object size. Based on such a result, it would often be concluded that grasping violated Weber's law (Ganel et al., 2008). However—as we have shown above—this conclusion would be premature. We first have to calculate ${\hat{JND}}_{\mathrm{MGA}}$ before we can assess Weber's law. To do this, we need to divide the SD_MGA_ by the local slope of the response function (computed from quadratic regression on individual participants) at that object size. These slopes are shown in Figure 3d.

After doing this, we found that ${\hat{JND}}_{\mathrm{MGA}}$ increased linearly with object size. The slope of this function corresponds to Weber's constant: *k*_MGA_ = 0.040 ± 0.013, *t*(19) = 3.18, *p* = 0.005, 95% CI, 0.014–0.066 (Figure 3e). This value of Weber's constant fits nicely within the expected range from the literature for size perception (0.02–0.06) (McKee & Welch, 1992; Teghtsoonian, 1971). Thus, when using an appropriate proxy for the JNDs, the uncertainty of the grasping response does increase with object size and thus follows Weber's law.

Finally, we checked whether grasping followed the well-known temporal pattern from the literature. Movement onset occurred 288 ± 1 ms after the goggles turned transparent. The movement duration (from onset until offset of the movement) was 293 ± 6 ms, and MGA was achieved on average 217 ± 8 ms after movement onset, such that MGA occurred at 71.2% to 76.6% of the movement duration, as expected from the literature (Jeannerod, 1984; Smeets & Brenner, 1999).

#### Manual estimation

The quadratic regressions revealed a curvilinear relationship between ME and object size (Figures 3a, 3b) (for regression coefficients, see Appendix C). The linear coefficient was *b*_ME_ = 1.05 ± 0.021, and the quadratic coefficient, *c*_ME_ = 0.0033 ± 0.001 mm^−1^, was different from zero. The residuals (Appendix B) show a systematic relationship, which disappears for the quadratic fit. *SD*_ME_ increased with object size, *b* = 0.056 ± 0.008, *t*(19) = 6.74, *p* < 0.001, 95% CI, 0.038–0.073 (Figure 3c). This finding is consistent with previous studies that also found such a scaling for ME. Based on such a result, it would often be concluded that manual estimation follows Weber's law (Ganel et al., 2008). But again, this conclusion would be premature. We first have to calculate $\hat{JND}$ before we can assess Weber's law. Again, we divided the *SD*_ME_ by the local slope (see Figure 3d) to calculate the $\hat{JND}$.

We found that ${\hat{JND}}_{\mathrm{ME}}$ increased with object size, resulting in a Weber constant of *k*_ME_ = 0.023 ± 0.014, *t*(19) = 1.65, *p* = 0.116, 95% CI, −0.006 to 0.051 (Figure 3e). We cannot claim that this result differs from zero, but crucially, *k* approached the typical magnitude expected for size perception (0.02 - 0.06; McKee & Welch, 1992; Teghtsoonian, 1971)*.* Given that we found clearly significant Weber's constant in manual estimation in the other studies we analyzed, and since researchers do not question that manual estimation adheres to Weber's law, we think this single non-significant result is no reason to question Weber's law in manual estimation.

### Preliminary summary

**Figure 4.**
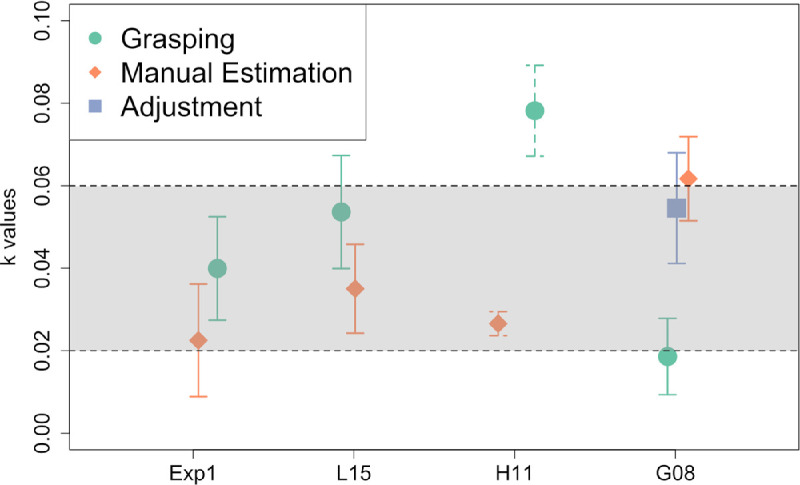
Weber constant *k* based on $\hat{JND}\;$for all of our analyses. From the literature, we expect *k* to be between 0.02 and 0.06 for visual size perception, as indicated by the shaded area. The *k* values we obtained are very close to this expected range. Values are shown as mean ± *SEM*. The values for H11 are dashed because we only had aggregate data for this study. Exp 1, Experiment 1; L15, Löwenkamp et al. (2015); H11, Heath et al. (2011); G08, Ganel et al. (2008).

We replicated the traditional results that are based on *SD*_Response_ as a proxy for JND. Based on this approach, manual estimation seems to follow Weber's law and grasping seems to violate it (Ganel et al., 2008). However, this conclusion would be premature because *SD*_Response_ is not a good proxy for JND when the response function is nonlinear. Therefore, we first have to calculate $\hat{JND}$ before we can assess Weber's law. When we do this, then grasping and manual estimation both seem to follow Weber's law, and the corresponding Weber constants *k* are in the range we would expect from the literature for size perception. This raises the question of how general our results are. To assess this, we reanalyzed three studies from the literature and calculated the Weber constants *k* based on $\hat{JND}$. For better comparison, we depicted all those Weber constants in Figure 4.

## Reanalysis of Löwenkamp et al. (2015)

The first study we reanalyzed was published by our own group (Löwenkamp et al., 2015) and served as a test case, because here all data were fully available (including trial-by-trial data), and all methodological details were known.

### Methods

Six objects (20, 30, 40, 50, 60, and 70 mm) were presented 20 times to 15 participants for grasping and to 15 different participants for manual estimation. Both grasping and manual estimation were performed open-loop. For further details, we refer to the original publication.

### Results and discussion

**Figure 5.**
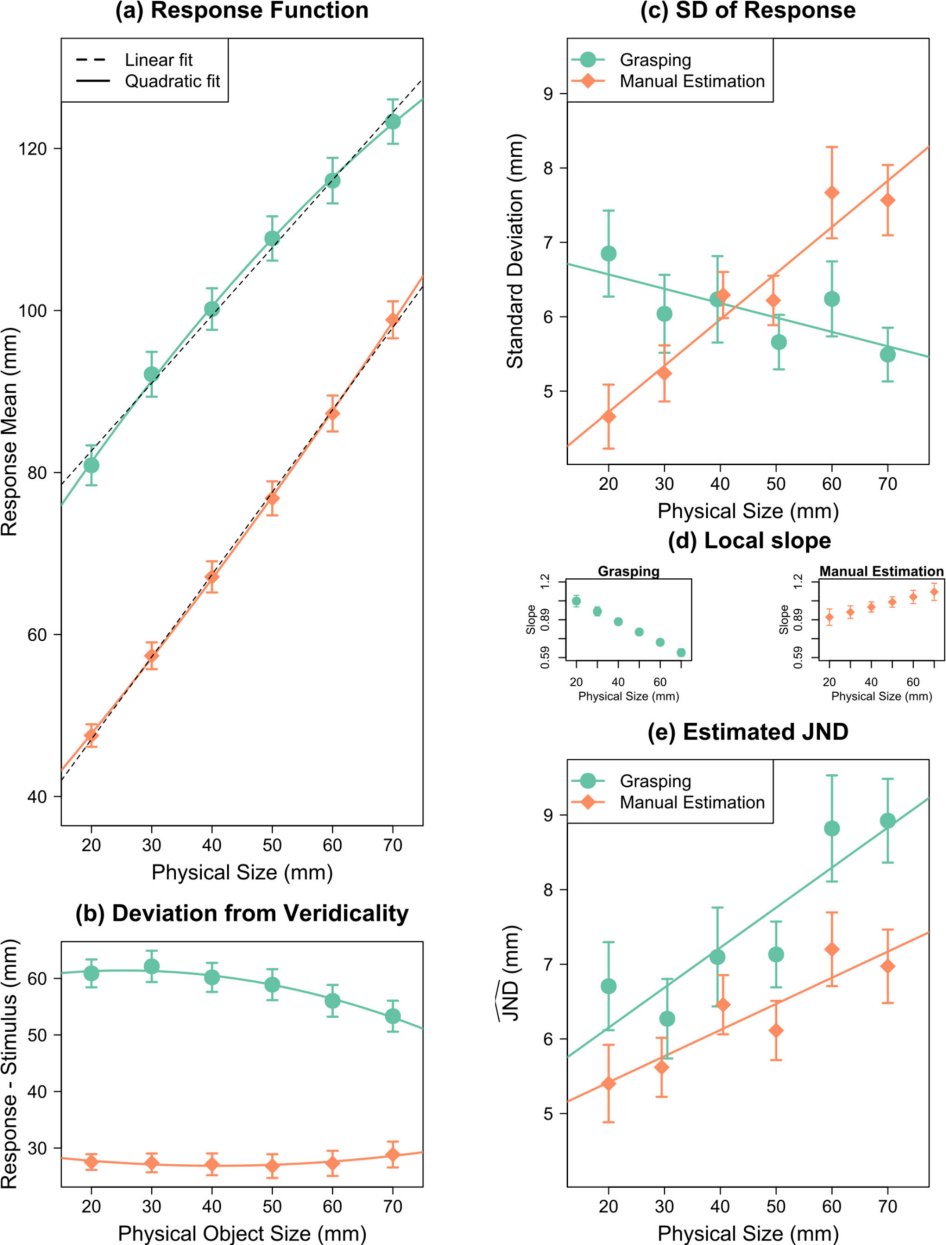
Reanalysis of Löwenkamp et al. (2015). (a) Mean responses as a function of object size. Quadratic regressions are shown as solid color curves; linear regressions as black dashed lines. (b) Difference between response (and quadratic fit) and physical stimulus size. The nonlinearity in the responses can be seen in the curvature of these differences. (c) *SD*_Response_ as a function of object size. (d) The local slope at every object size in the grasping and manual estimation. This is the value that the *SD* is divided by to calculate $\hat{JND}.$ (e) $\hat{JND}$ as a function of object size. Error bars depict ±1 *SEM* (between subjects).

Results are summarized in Figure 5, and regression coefficients are given in Appendix C. Figure 5a shows the response functions and Figure 5b deviations of the response from physical object size. The linear term *b* of the regression function for MGA was *b*_MGA_ = 0.84 ± 0.009 and for ME was *b*_ME_ = 1.02 ± 0.02. The quadratic term *c* was negative for MGA (*c*_MGA_ = −0.0042 ± 0.0003 mm^−1^), and positive for ME (*c*_ME_ = 0.0021 ± 0.0004 mm^−1^). As in Experiment 1, there was a concave relationship between MGA and object size (sign of *c* was negative); that is, the responsiveness of MGA decreased with increasing object size, whereas the responsiveness of ME changed much less.

*SD*_MGA_ did not scale significantly with object size, *b* = −0.019 ± 0.01, *t*(14) = −1.88, *p* = 0.08, 95% CI, −0.041 to 0.003 (Figure 5c), but *SD*_ME_ increased significantly with object size, *b* = 0.062 ± 0.007, *t*(14) = 8.56, *p* < 0.001, 95% CI, 0.046–0.078. Based on such a pattern of results, it would often be concluded that grasping violated Weber's law, whereas manual estimation followed Weber's law (Ganel et al., 2008). However, we again have to calculate $\hat{JND}$ first, before we can assess Weber's law.

In grasping, ${\hat{JND}}_{\mathrm{MGA}}$ increased significantly with object size, resulting in a Weber constant of *k*_MGA_ = 0.054 ± 0.014, *t*(14) = 3.91, *p* = 0.002, 95% CI, 0.024–0.083 (Figure 5e). Similarly, in manual estimation, ${\hat{JND}}_{\mathrm{ME}}\;\;$also increased significantly, with a Weber constant of *k*_ME_ = 0.035 ± 0.011, *t*(14) = 3.26, *p* = 0.006, 95% CI, 0.012–0.058. In short, grasping and manual estimation show Weber constants that are perfectly in the range we expect for size perception: 0.02 to 0.06 (McKee & Welch, 1992; Teghtsoonian, 1971) (Figure 4).

## Reanalysis of Heath et al. (2011)

Next, we investigated whether the data obtained in our own laboratory can be corroborated by data from other laboratories. For that, we first reanalyzed the data published by Heath et al. (2011). This was the only study we reanalyzed where only aggregated data (means across participants) were available (we tried without success to obtain the full data; see also Wicherts, Borsboom, Kats, & Molenaar, 2006). For the other studies (Experiment 1; Ganel et al., 2008; Löwenkamp et al., 2015) where we had full data, we made a comparison of our results with full versus aggregate data and found only minor differences. Therefore, we expect also only small differences to a full-data analysis of Heath et al. (2011). Given that we did not have access to the full data, the results might be slightly different when the participant-by-participant data are analyzed compared with those reported below with aggregated data. Because we are only able to roughly estimate the Weber's constant without having the full data, we do not show significance tests but only mean estimates with *SEM* and 95% confidence intervals.

### Methods

Five objects (20, 30, 40, 50, and 60 mm) were presented to 16 participants 20 times for grasping and to 11 participants 20 times for manual estimation. Manual estimation was performed under full-vision conditions. For grasping, we used the closed-loop mean values given in table 1 of Heath et al. (2011). Because the data had already been averaged over participants, our reanalysis was carried out at the level of the aggregated data. For further details on the experimental procedure, see the original publication.

### Results and discussion

**Figure 6.**
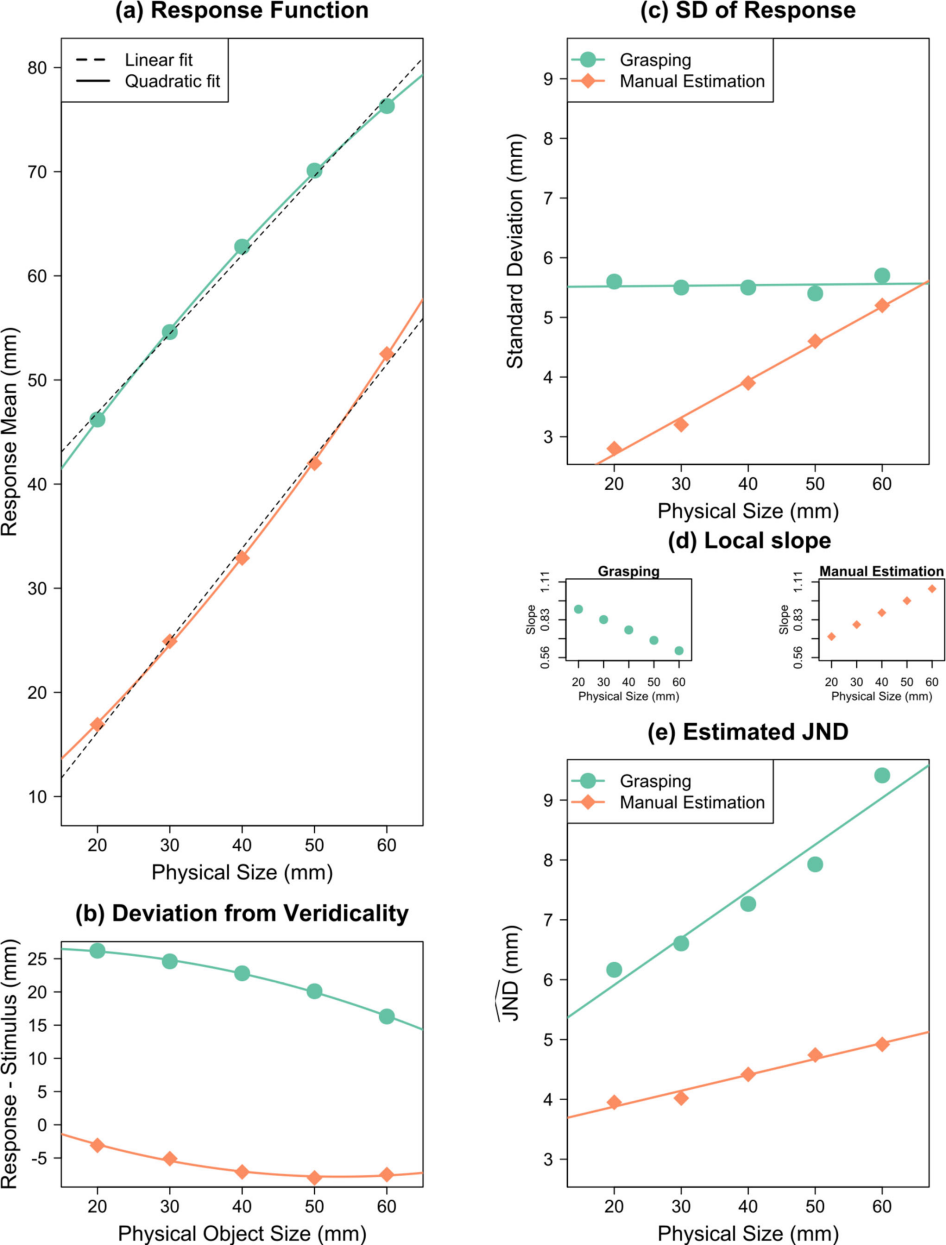
Reanalysis of Heath et al. (2011). (a) Mean responses as a function of object size. Quadratic regressions are shown as solid color curves; linear regressions as black dashed lines. (b) Difference between response (and quadratic fit) and physical stimulus size. The nonlinearity in the responses can be seen in the curvature of these differences. (c) *SD*_Response_ as a function of object size. (d) The local slope at every object size in grasping and manual estimation. This is the value that the *SD* is divided by to calculate $\hat{JND}.$ (e) $\hat{JND}$ as a function of object size. Error bars are absent because individual participant data were not available.

Results are summarized in Figure 6, and regression coefficients are given in Appendix C. Figure 6a shows the response functions, and Figure 6b shows the difference of the response from physical object size. The linear term *b* for MGA was *b*_MGA_ = 0.76 and for ME was *b*_ME_ = 0.88. The sign of the quadratic term *c* was negative for MGA (*c*_MGA_ = −0.0038 mm^−1^) and positive for ME (*c*_ME_ = 0.0044 mm^−1^).

*SD*_MGA_ (*b* = 0.001 ± 0.004; 95% CI, −0.012 to 0.014) did not change with object size (Figure 6c), whereas *SD*_ME_ increased with object size (*b* = 0.062 ± 0.003; 95% CI, 0.052–0.072), confirming the typically obtained result in such studies. Based on this pattern of results, it would often be concluded that manual estimation follows Weber's law but grasping does not (Ganel et al., 2008; Heath et al., 2011). But again, this conclusion would be premature. We first have to calculate $\hat{JND}$ before we can assess Weber's law.

In grasping, ${\hat{JND}}_{\mathrm{MGA}}$ increased with object size, resulting in a Weber constant of *k*_MGA_ = 0.078 ± 0.011 (95% CI, 0.043–0.113). In manual estimation,$\;{\hat{JND}}_{\mathrm{ME}}\;$also increased with object size, resulting in a Weber constant of *k*_ME_ = 0.027 ± 0.003 (95% CI, 0.017–0.036) (Figure 6e). These values again fit nicely with the expected range from the literature for size perception: 0.02 to 0.06 (McKee & Welch, 1992; Teghtsoonian, 1971) (Figure 4). In short, even for data from another laboratory (Heath et al., 2011), we find indications that grasping follows Weber's law if an appropriate proxy for JND is used. This raises the question of whether that is also true for the first, most influential landmark study on this topic (Ganel et al., 2008).

## Reanalysis of Ganel et al. (2008)

Finally, we applied our method to the data from the pioneering study on this subject (Ganel et al., 2008), which was the first to claim that grasping does not follow Weber's law.

### Methods

Six objects (20, 30, 40, 50, 60, and 70 mm) were presented 20 times to participants in grasping (*n* = 13), manual estimation (*n* = 11), and perceptual adjustment (i.e., adjustment of a comparison line on a monitor, the response to which we refer to as ADJ; *n* = 6). All tasks were performed in full-vision conditions. We obtained the data at the participant level from the authors. For further details on the experimental procedure, see the original publication.

### Results and discussion

**Figure 7.**
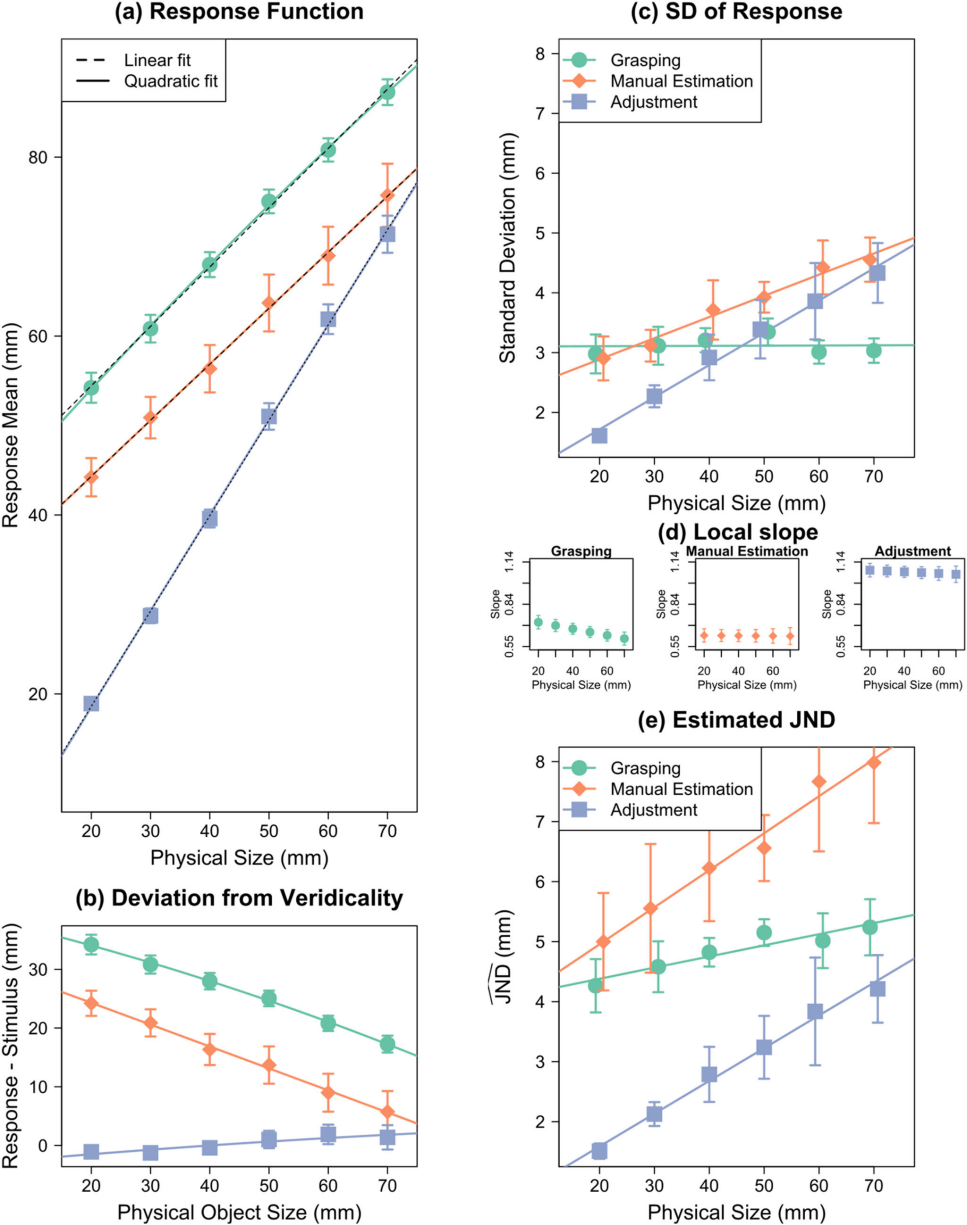
Reanalysis of Ganel et al. (2008). (a) Mean responses as a function of object size. Quadratic regressions are shown as solid color curves; linear regressions as black dashed lines. (b) Difference between response (and quadratic fit) and physical stimulus size. The nonlinearity in the responses can be seen in the curvature of these differences. (c) *SD*_Response_ as a function of object size. (d) The local slope at every object size in grasping, manual estimation, and adjustment. This is the value that the *SD* is divided by to calculate $\hat{JND}.$ (e) $\hat{JND}$ as a function of object size. Error bars depict ±1 *SEM* (between subjects).

Results are summarized in Figure 7, and regression coefficients are given in Appendix C. Figure 7a shows the response functions, and Figure 7b shows the difference between response and physical object size. The linear term *b* of the regression function for MGA was *b*_MGA_ = 0.66 ± 0.015; for ME, it was *b*_ME_ = 0.63 ± 0.16; and for ADJ it was *b*_ADJ_ = 1.07 ± 0.02. The quadratic term *c* for MGA was *c*_MGA_ = −0.0011 ± 0.0002 mm^−1^; for ME, it was *c*_ME_ = −0.00001 ± 0.0003 mm^−1^; and for ADJ, it was *c*_ADJ_ = −0.0003 ± 0.0003 mm^−1^ (see also Appendix C). It is interesting to note that the values of *c* for ME and ADJ are very close to zero, because the response function is almost perfectly linear (we will further discuss this below).

The *SD*_MGA_, linear term *b* = 0.0003 ± 0.004, *t*(12) = 0.078, *p* = 0.939, 95% CI, −0.008 to 0.009, did not scale with object size, whereas *SD*_ME_, *b* = 0.035 ± 0.005, *t*(10) = 7.23, *p* < 0.001, 95% CI, 0.025–0.046, and *SD*_ADJ_, *b* = 0.054 ± 0.01, *t*(5) = 5.46, *p* = 0.003, 95% CI, 0.029–0.079, scaled with object size (Figure 7c). This was interpreted as an absence of Weber's law in grasping by Ganel et al. (2008). However, as we have shown, conclusions about Weber's law should only be made after calculating the $\hat{JND}s.$

The ${\hat{JND}}_{\mathrm{MGA}}$, ${\hat{JND}}_{\mathrm{ME}}$, and the ${\hat{JND}}_{\mathrm{ADJ}}$ all increased linearly with object size (Figure 7e). The values of the Weber constant *k* for MGA, *k*_MGA_ = 0.019 ± 0.009, *t*(12) = 2.01, *p* = 0.068, 95% CI, −0.002 to 0.039, ME, *k*_ME_ = 0.062 ± 0.010, *t*(10) = 6.05, *p* < 0.001, 95% CI, 0.039–0.084, and ADJ, *k*_ADJ_ = 0.055 ± 0.013, *t*(5) = 4.07, *p* = 0.01, 95% CI, 0.020–0.089, were again consistent with the expected values from the literature on size perception: 0.02 to 0.06 (McKee & Welch, 1992; Teghtsoonian, 1971) (Figure 4). The Weber's constant in grasping did not reach significance; however, the value was in the expected range. Also, the 95% CI overlapped with the expected range of *k.* Further, in the three other studies we analyzed, the Weber's constant in grasping was highly significant. Ganel et al. (2008) had only 13 participants, which may have been too few, leading to low power. Overall and based on the results from all studies, we conclude that there is evidence for grasping following Weber's law.

## General discussion

Research on Weber's law in grasping has typically used the response variability (*SD*_MGA_) as a proxy for JND—starting with Ganel et al. (2008). We showed that this were only acceptable if the response function were linear. If, however, the response function is nonlinear—as is the case in grasping—then we first have to transform the response variability back to the corresponding stimulus variability (Figure 1). That is, we have to transform the hitherto used *SD*_MGA_ to corresponding $\hat{JND}s$ (Appendix A). Only then does it make sense to assess Weber's law.

Using this toolkit, we first analyzed our own data of Experiment 1 and showed that we were able to replicate the results for grasping: *SD*_MGA_ does not increase with object size. Traditionally, this result would have been interpreted as a violation of Weber's law (Ganel et al., 2008); however, the response function of MGA in grasping is nonlinear. It is concave, such that responsiveness decreases for larger object sizes (one reason could be because the finger span is limited and grasping needs to trade-off a safety margin with the ability to still enclose the target object). Therefore, it is not appropriate to use *SD*_MGA_ as a proxy for JND. Instead, we need to transform *SD*_MGA_ back to the corresponding variability at the stimulus level. When we do this and calculate ${\hat{JND}}_{\mathrm{MGA}}$, then we find that ${\hat{JND}}_{\mathrm{MGA}}$ does increase with object size. The corresponding Weber constant *k* (i.e., the slope of the linear function relating ${\hat{JND}}_{\mathrm{MGA}}$ to object size) is perfectly in the range we would expect from the literature for size perception (Figure 4).

Next, we used the same toolkit to reanalyze the data of three already published studies that tested Weber's law for grasping, including the landmark study by Ganel et al. (2008). In all cases, we find similar results for grasping: (a) *SD*_MGA_ does not increase with object size; (b) the response function of MGA in grasping is nonlinear; and (c) $\hat{JND}\;$seems to increase with object size and the corresponding Weber constant is well in the expected range (Figure 4). We conclude that there is evidence for Weber's law in grasping.

Tasks other than grasping have been employed, most notably manual estimation. We calculated ${\hat{JND}}_{\mathrm{ME}}$, as described above for grasping, and the corresponding Weber constants and found them again to be in the expected range and similar to the Weber constants for grasping (Figure 4). We conclude that there seems to be no dissociation among grasping, manual estimation, and other measures regarding Weber's law.

What are the consequences for theorizing? First of all, there is a coherent picture of this psychophysical law again: Researchers can trust that Weber's law, the first and most widely tested psychophysical principle (Baird & Noma, 1978; Ganel et al., 2008), is almost universally correct (Teghtsoonian, 1971). Second, there is a chance that the wide and contradictory literature that was inspired by the claim of a violation of Weber's law in grasping (Ayala et al., 2018; Christiansen et al., 2014; Freud et al., 2019; Ganel et al., 2008; Hadad et al., 2012; Heath & Manzone, 2017; Heath et al., 2011; Heath et al., 2012; Heath et al., 2017; Holmes et al., 2011; Holmes & Heath, 2013; Hosang et al., 2016; Jazi & Heath, 2017; Löwenkamp et al., 2015; Namdar et al., 2018; Ozana & Ganel, 2017; Ozana & Ganel, 2018; Ozana et al., 2018; Utz et al., 2015) might be rectified to a coherent view again. In the following, we will first discuss details of our approach and then specific consequences for theorizing about information processing in the brain.

### Further details related to Weber's law in grasping and manual estimation

There are some more technical issues related to our arguments and approach that we want to address.

#### What happens when IQR is used as proxy for JND?

Some studies on Weber's law in grasping (Bruno et al., 2016; Utz et al., 2015) used the interquartile range (*IQR*_Response_) or similar measures of dispersion to assess Weber's law instead of the most often used within-subject standard deviation (*SD*_Response_). The main motivation was the well-established fact that these measures are more robust against extreme values and outliers than the *SD*_Response_. However, our arguments apply equally to these alternative measures because they still quantify the variability at the level of the response, not the stimulus. Due to the nonlinear response function in grasping, it is therefore still possible for the *IQR*_Response_ to not scale with object size, whereas the corresponding $\hat{JND}$ does (the arguments are analogous to those we present for *SD*_Response_ in Figure 1). Therefore, even when *IQR*_Response_ is used, it should be divided by the local slope at every object size to obtain the corresponding $\hat{JND}$.

In the present study, we focused on *SD*_Response_ because this is the measure that was used by studies inferring a strong violation of Weber's law in grasping. Studies based on *IQR*_Response_ or other alternative measures typically favored the biomechanical constraints approach which is consistent with our main conclusions and which we discuss below.

#### Studies on other topics also used *SD* as a proxy for JND. Are they all wrong?

Weber's law has been demonstrated in almost every sensory domain (Teghtsoonian, 1971). Some studies that reported Weber's law in domains other than grasping also used *SD*_Response_ for this assessment (for a list, see Ganel, Freud, & Meiran, 2014). The question now arises whether all those studies need to be reanalyzed by calculating the $\hat{JND}$s instead. This would require dividing at each stimulus magnitude the *SD*_Response_ by the local slope of the response function (i.e., the function relating stimulus magnitude to response).

Fortunately, such a major undertaking does not seem necessary. In most cases, the response function will be linear, such that the local slope is constant for each stimulus magnitude. That is, the *SD*_Response_ would at each stimulus magnitude be divided by the same constant value, such that $\hat{JND}=\;\frac{SD_{Response}}{Constant}$. This constant scaling of *SD*_Response_ will not change the assessment of whether the $\hat{JND}$s scale with object size, such that the answer to the question of whether the sensory domain adheres to Weber's law will not change. In many cases, the slopes will even be close to 1, such that *SD*_Response_ ≈ $\hat{JND}$ so that both measures will even give the same numerical answer (e.g., the perceptual adjustment task of Ganel et al., 2008) (see Figure 7d). However, in situations where the response function clearly deviates from linearity, as is the case for grasping, the *SD*_Response_ is not appropriate for assessing Weber's law, and ${\hat{JND}}_{\mathrm{Response}}$ must be calculated.

#### Should the function relating JND to object size have a zero intercept?

Researchers investigating Weber's law typically test for a linear relationship between stimulus magnitude and JND; that is, they allow for a non-zero intercept of the linear function relating stimulus magnitude to JND (e.g., equation 4.1 of Baird & Noma, 1978; Brown et al., 1962; Miller, 1947; von Helmholtz, 1924). This is often referred to as the *generalized* *version* of Weber's law (Miller, 1947; Ono, 1967). This is also the approach we used, and our results are nicely consistent with the literature (Figure 4). However, the *strict version* of Weber's law predicts a proportional relationship between stimulus magnitude and JND—that is, a linear function with a zero-intercept (e.g., equation 3.1 of Baird & Noma, 1978). Therefore, the question arises whether it would be better to use this strict version of Weber's law on the current data.

We will discuss this issue in two strands: First, we will argue that the large psychophysical literature on Weber's law in different sensory domains is often based on the generalized version of Weber's law, such that for comparison with this literature we need to use the generalized version. Second, we will show that Weber's law holds for the current data even if we assumed a strict version of Weber's law with zero-intercept. In fact, the estimated Weber constants would even increase when using such a strict version of Weber's law; that is, we are being conservative with respect to our main result that grasping follows Weber's law when we use the generalized Weber's law.

So, let us first point out that the psychophysical literature often uses the generalized version of Weber's law. For example, Teghtsoonian (1971) summarized studies on size perception in a seminal review and used the generalized Weber's law, thereby allowing for a non-zero intercept. Also, we know that other tasks and modalities show similar non-zero intercepts and that the strict version of Weber's law does not provide good fits (Baird & Noma, 1978; Brown et al., 1962; Miller, 1947; Ono, 1967; von Helmholtz, 1924). Therefore, using the generalized version of Weber's law is not problematic for the claim that grasping or manual estimation are consistent with Weber's law. Specifically, if allowing for a non-zero intercept was considered to be an argument against Weber's law in grasping, then the same argument could also be used against Weber's law in manual estimation because manual estimation also shows a non-zero intercept. Similarly, if the non-zero intercept were considered to be an argument against our method of calculating $\hat{JND}$, then the same argument could be used against the traditional method that uses *SD*_Response_ as a proxy for JND, because this method also finds non-zero intercepts for grasping and manual estimation (Ganel et al., 2008; Heath et al., 2011) (see also Appendix C).

Second, let us describe the empirical results when using the strict version of Weber's law. This strict version comes in two variants. In Appendix E1, we show the results when fitting linear models with zero intercept. The obtained Weber constants (Table E1) are much larger than those obtained with the generalized version of Weber's law (Figure 4). In Appendix E2, we use an alternative analysis that was suggested by a reviewer and is based on Smeets & Brenner (2008). The Weber constants attained by this method are also larger (Table E2) than those attained with the generalized version of Weber's law (Figure 4). This shows that we are being conservative with respect to Weber's law in grasping when we use the generalized version of Weber's law. Any of the proposed strict versions of Weber's law would yield larger Weber constants. The fact that we used the most conservative analysis makes our finding of Weber's law in grasping even stronger.

#### Technical details regarding nonlinear calculation of $\hat{JND}s$

To estimate the JND, the within-participant *SD* at each object size for each participant should be divided by the local slope (see each panel d in Figures 3 and 5,^6^–7) of the response function (for that participant) at that object size. Essentially, the $\hat{JND\;}$ is a ratio with the measured slope in the denominator. If the measured slope for any participant is a small value close to zero, this can lead to inflated values (and variability) in the final $\hat{JND\;}$. This problem of ratios is well known from statistical calibration (Buonaccorsi, 2001), and there also exist methods to ameliorate this problem (Franz, 2007; von Luxburg & Franz, 2009). One reason why measured slopes may be close to zero is not having enough trials (approximately <20 trials per object size). Therefore, researchers applying this method to their own data and other data need to be careful that there were sufficient trials per object size. In Experiment 1, we used 50 trials per object size, and we reanalyzed only those studies that had 20 or more trials per object size.

### Consequences for theories about information processing in the brain

As mentioned above, the reports of an apparent violation of Weber's law generated a number of different explanations. Given that we found Weber's law to not be violated in grasping when the $\hat{JND}s$ are assessed, we will discuss the consequences of our findings for these explanations.

#### Perception–action model: Are there two parallel visual processing streams?

Ganel et al. (2008) interpreted the presumed violation of Weber's law in grasping as strong evidence for the perception–action model. This model assumes that there are fundamental and qualitative differences in the neural processing of size information for the purposes of action and perception (Goodale, 2008; Goodale, 2011; Goodale & Milner, 1992; Milner & Goodale, 1995; Milner & Goodale, 2008). According to the perception–action model, visual information used for perception (such as in perceptual estimations, but also certain “perceptual” actions as manual estimation) is processed in the ventral cortical pathway and is based on relative metrics. In contrast, visual information used in natural actions (such as in natural grasping), is assumed to be processed in the dorsal cortical pathway and based on absolute metrics. The apparent violation of Weber's law in grasping was seen as one line of evidence for such a strict division of labor in the brain (Ganel et al., 2008; Ganel et al., 2014). However, the results of the present study suggest that grasping does follow Weber's law. Accordingly, there is no need to postulate differences in the visual encoding of size information between grasping and manual estimation, as suggested by Ganel et al. (2008). This calls into question another line of evidence that has been put forward in support of the perception–action model, as have been other lines of evidence (e.g., Brenner & Smeets, 1996; de la Malla, Brenner, de Haan, & Smeets, 2019; Franz & Gegenfurtner, 2008; Franz, Gegenfurtner, Bülthoff, & Fahle, 2000; Hesse & Schenk, 2014; Kopiske, Bruno, Hesse, Schenk, & Franz, 2016; Medendorp, de Brouwer, & Smeets, 2018; Schenk, 2006; Smeets & Brenner, 1995; Smeets, Kleijn, van der Meijden, & Brenner, 2020).

#### Double-pointing hypothesis: Is grasping guided by position and not size?

An alternative explanation for the presumed absence of Weber's law in grasping has been proposed by Smeets & Brenner (2008). They argued that grasping does not follow Weber's law because grasping uses position information about the contact points of the fingers on the object instead of size information (Smeets & Brenner, 1999; Smeets, van der Kooij, & Brenner, 2019; Smeets et al., 2020). That is, the fingers are moved individually to the contact points on the object such that object size is not used; consequently, researchers cannot expect Weber's law in grasping when object size is manipulated. However, given that we do find Weber's law in grasping, this alternative explanation for an apparent absence of Weber's law is no longer relevant.

We should stress, however, that this state of affairs does not need to be an argument against the double-pointing hypothesis itself. This is so because the presence or absence of Weber's law is not necessarily considered to be a strong test case for the double-pointing hypothesis. Notwithstanding that, there is one question that arises from the current results and that might be interesting for future research on grasping and the double-pointing hypothesis: Smeets & Brenner (1999) showed that the double-pointing hypothesis predicts a small, positive quadratic term for the relationship between MGA and object size; that is, the responsiveness in grasping should increase with object size. However, we found that the quadratic terms in grasping were slightly negative. This apparent contradiction should be investigated in future research—as well as the more general question of whether grasping is guided by position information, size information, or a mixture of both. For examples of such research, see Schot et al. (2017) on prism adaptation or Smeets et al. (2020) on certain visual illusions.

#### Boundary conditions: When is Weber's law violated in grasping?

A number of studies assessed the boundary conditions for a presumed violation of Weber's law in grasping. Typically, the idea was to learn something about the mechanisms that cause the presumed violation of Weber's law. These studies investigated, for example, whether grasping adhered to Weber's law when the object is shown only in 2D (Hosang et al., 2016), when the object is distorted by certain visual illusions which selectively distort the size of the object but not the positions of the grasp points on the object (Smeets et al., 2020), in pantomimed grasping (Jazi & Heath, 2016; Jazi & Heath, 2017; Jazi, Yau, Westwood, & Heath, 2015), when grasping 3D objects underneath a glass surface (Ozana & Ganel, 2017), or when grasping with both hands in a virtual environment (Ozana, Berman, & Ganel, 2020). Some studies (Holmes et al., 2011) also looked at the time course and development of Weber's law over the entire grasping trajectory (but see Foster & Franz, 2013). However, all of these studies relied on *SD*_MGA_ as a proxy for JND; therefore, it is difficult to judge the results. For a full assessment, we would need to transfer *SD*_MGA_ to $\hat{JND}$ first. This is all the more important as it is plausible that those manipulations change not only the variability of the response but also the response function, in one or the other way (larger or smaller safety margin, depending on the specific manipulation). For example, it is well known that the safety margin of MGA is smaller in 2D and pantomimed grasping, conditions where no object is physically grasped. Weber's law should then be assessed by calculating the $\hat{JND}s$.

#### Biomechanical constraints and ceiling effects in the motor response

Biomechanical constraints (e.g., limited finger span) have been proposed as another alternative explanation for the presumed violation of Weber's law in grasping (Löwenkamp et al., 2015; Utz et al., 2015). These studies also investigated the response function in detail and found that for larger object sizes the response function is bent (consistent with what we found in the present study). They argued that for large objects the fingers simply cannot open wider and that this will automatically restrict the variability of grasping. Therefore, so they argue, it is plausible that Weber's law is masked by these biomechanical constraints in grasping and that there is no need to postulate a violation of Weber's law based on the fact that *SD*_MGA_ does not increase with object size.

Those studies and their interpretation corroborate our current findings. They also have found that the response function is bent and provide a plausible mechanism for why *SD*_MGA_ does not increase with object size. The difference to the present study is that we now have a toolkit that allows us to quantitatively assess whether Weber's law does or does not hold, *independent* of the exact mechanisms that cause the response function to be bent. It is important to note here that our results do not rest upon biomechanical constraints being the reason for nonlinearity in the grasping response function—it is just one plausible mechanism that has been discussed by others in the literature.

Interestingly, we found some evidence of such effects in Experiment 1, as the skewness of the response distributions scaled negatively with object size (i.e., the frequency of relatively large responses decreased with object size) in grasping (*b*_skew_ = −0.0085 ± 0.003; 95% CI, −0.015 to −0.002), but not in manual estimation (*b*_skew_ = −0.0037 ± 0.004; 95% CI, −0.012 to 0.005). Note that we found this result even within a range of medium objects (i.e., 20–50 mm in Experiment 1; see also Löwenkamp et al., 2015), which have been termed “functionally graspable” and for which it was sometimes assumed that the influence of biomechanical constraints can be excluded (Ayala et al., 2018; Heath et al., 2017). Nevertheless, it seems plausible that optimization processes in the generation of the MGA, such as the generation of comfortable or efficient grip apertures, may cause these effects even at small, graspable object sizes. A recent study (Uccelli et al., 2021) assessed Weber's law in small-to-medium object sizes (5–40 mm) and reported that the skewness of the MGA increased with object size up to 40 mm. In a previous study by the same group (Bruno et al., 2016), where stimuli larger than 40 mm were also used, small effects for a negative scaling of skewness at objects larger than 40 mm were found. We agree with those authors’ conclusion that a study investigating the skewness of the MGA in the full range of object sizes up to the limit of the handspan would be useful to make definite claims (Uccelli et al., 2021). It is possible that the skewness follows an inverse-U–shaped function of object size.

**Figure 8.**
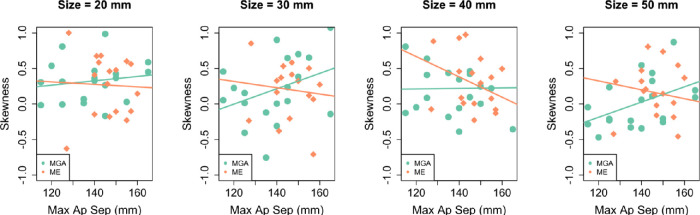
Skewness as a function of hand size. The skewness in the response of every participant at every object size is plotted as a function of the hand size or the maximum aperture separation (MAS) of that participant. Also depicted are the regression lines for skewness as a function of MAS.

Additionally, biomechanical constraints will not affect all participants equally but rather will depend on the hand size and maximum opening in relation to the target object size. To investigate this, we analyzed the skewness at every object size as a function of the participants’ hand size (maximum aperture separation [MAS]) (Figure 8). For our largest object size (50 mm), we found that participants’ skewness increased with hand size in grasping (*b*_skew_ = 0.011 ± 0.005; 95% CI, 0.000–0.021, with MAS values for 19 out of 20 participants in grasping). This scaling of skewness with hand size was negligible at our smallest object size (20 mm).

#### Apparent inversion of Weber's law: A puzzle for certain theories

Several studies (Bruno et al., 2016; Löwenkamp et al., 2015; Utz et al., 2015) reported a puzzling result of *decreasing* variability in the MGA with object size, or an apparent *inversion* of Weber's law in grasping. This result was especially confusing, because none of the theories proposed to explain the absence of Weber's law in grasping (perception–action model or double-pointing hypothesis) could accommodate this finding. Utz et al. (2015) reasoned that biomechanical constraints on the finger aperture could cause ceiling effects in grasping large objects. These constraints combined with a nonlinear grasping response function can readily explain this strange result of apparently inverted Weber's law. When grasping a large object, the finger aperture will be larger than the to-be-grasped object (including the safety margin), and this is capped by the maximum possible opening of the hand. As object sizes increase after the point where object size + safety margin is close to the maximum possible hand opening, the safety margin will decrease in compensation, thus leading to decreasing variability in the response. When we reanalyzed Löwenkamp et al. (2015), the apparent inversion of Weber's law disappeared and the $\hat{JND}$ scaled positively with object size, as expected by Weber's law, and, notably, with values of Weber's constant *k* (slope) consistent with the literature (Figure 4). Therefore, our approach can readily explain these inconsistencies in the large literature on Weber's law in grasping.

### Influences of sensory or motor noise

One might ask how motor noise influences the responses in grasping and manual estimation. Could it be that the variability generated during the movement overwrites the variability of some internal estimate that was used to generate those movements? We will argue that this question is interesting, but not so relevant for our investigation. This is so because Weber's law describes a relationship between the physical stimulus magnitude and the JND, the physical change in the stimulus that is detectable by the participant. Therefore, everything is at the level of the physical stimulus, and discussion of internal estimates is not required or crucial for Weber's law. We merely found that grasping does indeed follow Weber's law. The question of internal estimates would be more urgent if we did not find Weber's law but suspected that, for example, the variability of some internal estimate scaled nevertheless with object size (even when the JNDs did not). Here we simply establish that, contrary to previous research, grasping does follow Weber's law. Determining the contribution of sensory or motor noise to this Weberian scaling of JNDs in grasping is an undertaking for future research.

## Conclusions

Weber's law is one of the most fundamental psychophysical principles. It relates JND to stimulus magnitude and therefore makes a statement at the level of the physical stimuli. Studies reporting a violation of Weber's law for grasping used *SD*_MGA_ as proxy for JND and therefore forsook the stimulus level. We showed that this is problematic when the response function is nonlinear (as in grasping) and that instead $\hat{JND}$ must be calculated, which brings us back to the stimulus level. If we do this, then grasping follows Weber's law—in our own data, as well as in previous studies that claimed a violation of Weber's law for grasping. Our method is general and can also be used in other tasks and sensory domains whenever a direct assessment of JND is not possible.

## Figures and Tables

**Figure A1.**
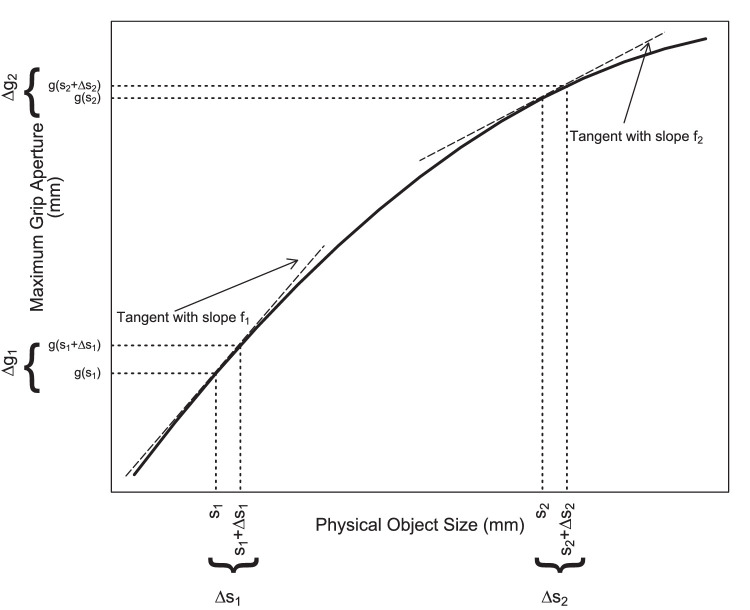
Concave (“bent”) grasping response function *g*(*s*).

**Figure B1.**
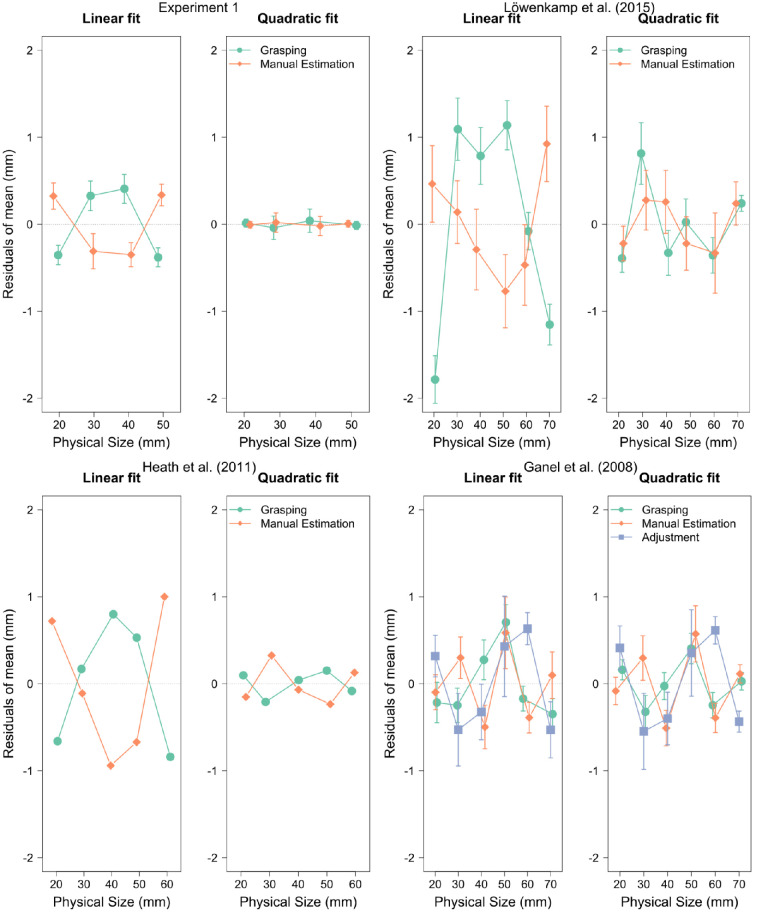
Residuals of the mean in the linear and quadratic regression functions for each dataset. The error bars depict ±1 RSE, where full participant data were available. RSE = residual standard errors.

## Footnotes

1
Ganel et al. (2008) worked within the framework of the perception–action model, which considers certain tasks as being driven by “perception” and other tasks as being driven by “action” (see General discussion for details). For ease of exposition, we will follow this labeling, albeit it could be questioned whether, for example, manual estimation is truly only a “perceptual” task (e.g., Franz, 2003).

## Appendix A Appendix A. How to determine $\hat{JND}$ from *SD*_MGA_

In the main text, we have argued that *SD*_MGA_ is not an appropriate proxy for the corresponding JND and that we have to use $\hat{JND}$ instead. Here, we describe how to calculate $\hat{JND}$. For ease of exposition, we will derive our formulae for the special case of grasping, albeit all formulae can directly be generalized to other responses (for example, manual estimation or size adjustment). Our scenario is shown in Figure A1 (which is similar to Figure 1 of the main text but contains more technical details). A participant is presented with objects of different sizes *s*. When the participant grasps those objects, their sizes are transferred to MGAs by the response function *g*(*s*). For simplicity of exposition, we focus on the stimulus with physical size *s*_1_.

### Forward direction

Here, we are interested in the effect an increase of object size by a small amount has on grasping (this small amount could, for example, be one JND). For this, assume that the participant grasped first an object of size *s*_1_, resulting in an MGA of *g*(*s*_1_). We now increase the size of the object by a small amount (which we denote as ∆*s*_1_), such that the second object has the size *s*_1_ + Δ*s*_1_, resulting in an MGA of *g*(*s*_1_ + ∆*s*_1_). The effect of increasing the size of the object by ∆*s*_1_ on MGA in grasping is then

(1) $$\begin{array}{c} \Delta g_{1}=g\left(s_{1}+\Delta s_{1}\right)-g\left(s_{1}\right)\; \end{array}$$

Now, we want to find a simple expression for this effect. We perform a first-order Taylor series approximation:

(2) $$\begin{array}{ccc} g\left(s_{1}+\Delta s_{1}\right) & \;= & g\left(s_{1}\right)+\frac{dg}{ds}{|}_{s_{1}}\Delta s_{1}\\ & & \;+\;\mathrm{higher}\;\mathrm{order}\;\mathrm{terms}\; \end{array}$$

(3) $$\begin{array}{ccc} & & \approx g\left(s_{1}\right)+\frac{dg}{ds}{|}_{s_{1}}\Delta s_{1}\; \end{array}$$

and combine this approximation with Equation 1:

(4) $$\begin{array}{c} \Delta g_{1}\approx g\left(s_{1}\right)+\;\frac{dg}{ds}{|}_{s_{1}}\Delta s_{1}-g\left(s_{1}\right)=\frac{dg}{ds}{|}_{s_{1}}\Delta s_{1}\; \end{array}$$

For simplicity, we rename $\frac{dg}{ds}{|}_{s_{1}}$, which is the slope of the tangent to *g*(*s*) at the position *s*_1_, as $f_{1}:=\;\frac{dg}{ds}{|}_{s_{1}}$, such that we obtain

(5) $$\begin{array}{c} \Delta g_{1}\;\approx f_{1}\Delta s_{1}\; \end{array}$$

This is the main result for the forward direction. When we increase object size by a small amount ∆*s*_1_, then MGA is increased by this amount times the slope *f*_1_ (the first derivative) of the response function *g*(*s*) that relates object size to MGA.

Some comments might be helpful here. By a small amount we, of course, mean an increase of object size for which the local linear approximation of the Taylor series is satisfactory. Given that we are interested in JNDs and that the response functions show only relatively small nonlinearities, this is a reasonable approximation. Also, because Equation 5 holds for any small size change, we can directly infer that this will also hold for the JNDs, as well as for related *SD*s, such that

(6) $$\begin{array}{c} SD_{\mathrm{MG}A_{1}}\approx f_{1}\;{\hat{JND}}_{1}\; \end{array}$$

### Backward direction

Now we are interested in the inverse problem: We observe some small change in the MGA of grasping ∆*g*_1_ and want to know to which change in object size ∆*s*_1_ this corresponds. Given our local linear approximation, this is now easy to do. We only need to invert Equation 5 such that we obtain

(7) $$\begin{array}{c} \Delta s_{1}\approx \frac{\Delta g_{1}}{f_{1}}\; \end{array}$$

Because the factor *f*_1_ in this equation is constant for any small size change, we can directly infer which $\hat{JND}$ corresponds to a *SD*_MGA_:

(8) $$\begin{array}{c} \hat{JND_{1}}\approx \frac{SD_{\mathrm{MG}A_{1}}}{f_{1}}\; \end{array}$$

This is the main result for the backward direction. When we measure the standard deviation of grasping *SD*_MGA_ and want to know to which standard deviation this corresponds at the level of physical object size, we just need to divide *SD*_MGA_ by the slope *f*_1_ (the first derivative) of the response function *g*(*s*) that relates object size to MGA.

### Application to our datasets

The above derivations are valid for quite arbitrary nonlinear relationships and are therefore very general (only limited by the applicability of the Taylor series approximation). In the practical application to the data we were concerned with, the nonlinear relationship was even simpler, because it was dominated by the linear and quadratic terms. This allowed us to simplify the relationships even further by fitting quadratic functions to the data:

(9) $$\begin{array}{c} g\left(s\right)=a+bs+cs^{2}\; \end{array}$$

where we used a standard least-squares approach for fitting. Of course, all the above derived relationships are also valid in this simpler case. One advantage of the quadratic fit is that we can derive an analytic expression for the scale factor, such that

(10) $$\begin{array}{c} f_{1}=\frac{dg}{ds}{|}_{s_{1}}=b+2cs_{1}\; \end{array}$$

This gives us for the calculation of the proxy for JND for a stimulus of size *s*_1_:

(11) $$\begin{array}{c} \hat{JND_{1}}\;\approx \frac{SD_{\mathrm{MG}A_{1}}}{f_{1}}=\frac{SD_{\mathrm{MG}A_{1}}}{b+2cs_{1}}\; \end{array}$$

The residuals of the linear and quadratic fits are shown in Appendix B; numerical values of all fitted coefficients are given in Appendix C.

### Assessment of Weber's law

After determining $\hat{JND}$ for each stimulus size, we were able to assess Weber's law. That is, we tested whether $\hat{JND}$ depends on stimulus size. For this, we fitted linear functions:

(12) $$\begin{array}{c} \hat{JND}\left(s\right)=a+ks\; \end{array}$$

where we again used a standard least-squares approach for fitting. The crucial parameter here is *k*, which indicates to which degree $\hat{JND}$ increases with object size, as predicted by Weber's law. This parameter is traditionally referred to as the Weber constant or Weber fraction. Numerical values for all these fitted coefficients are also given in Appendix C.

## Appendix B. Residuals of linear and quadratic fit

Residuals of the mean in the linear and quadratic regression functions for each dataset are shown. The error bars depict ±1 residual standard error (*RSE*), where full participant data were available.

## Appendix C. Coefficients for linear and quadratic regressions

**Table C1.** Coefficients of linear and quadratic regressions for maximum grip aperture (MGA), manual estimation (ME), and adjusted size (ADJ). The coefficients of quadratic regression relating object size to response (*a* and *b* of linear and quadratic regression) are identical because object size was centered. *SD*, coefficient of linear regression relating object size to *SD*_Response_; $\hat{JND}$, coefficient of linear regression relating object size to $\hat{JND}$. For details, see Appendix A.

		
				Coefficients (mean ± *SEM*) for reanalysis of								
	Experiment 1 coefficients (mean ± *SEM*)			Löwenkamp et al. (2015)			Heath et al. (2011)			Ganel et al. (2008)		
Dataset	Intercept (mm)	Linear	Quadratic (mm^−1^)	Intercept (mm)	Linear	Quadratic (mm^−1^)	Intercept (mm)	Linear	Quadratic (mm^−1^)	Intercept (mm)	Linear	Quadratic (mm^−1^)
MGA	*a* = 76.78 ± 0.56	*b* = 1.04 ± 0.013	*c* = −0.0037 ± 0.001	*a* = 104.79 ± 1.08	*b* = 0.84 ± 0.009	*c* = −0.0042 ± 0.0003	*a* = 62.76	*b* = 0.76	*c* = −0.0038	*a* = 71.36 ± 0.53	*b* = 0.66 ± 0.015	*c* = −0.0011 ± 0.0002
*SD*_MGA_	*a* = 5.25 ± 0.50	*b* = 0.002 ± 0.010	—	*a* = 6.96 ± 0.71	*b* = −0.019 ± 0.010	—	*a* = 5.5 ± 0.18	*b* = 0.001 ± 0.004	—	*a* = 3.1 ± 0.34	*b* = 0.0003 ± 0.004	—
${\hat{JND}}_{\mathrm{MGA}}$	*a* = 3.79 ± 0.47	*k* = 0.040 ± 0.013	—	*a* = 5.08 ± 0.75	*k* = 0.054 ± 0.014	—	*a* = 4.35 ± 0.47	*k* = 0.078 ± 0.011	—	*a* = 4.01 ± 0.48	*k* = 0.019 ± 0.009	—
ME	*a* = 59.68 ± 1.17	*b* = 1.05 ± 0.021	*c* = 0.0033 ± 0.001	*a* = 71.90 ± 0.78	*b* = 1.02 ± 0.02	*c* = 0.0021 ± 0.0004	*a* = 32.97	*b* = 0.88	*c* = 0.0044	*a* = 59.99 ± 1.12	*b* = 0.63 ± 0.16	*c* = −0.00005 ± 0.0003
*SD*_ME_	*a* = 3.12 ± 0.35	*b* = 0.056 ± 0.008	—	*a* = 3.48 ± 0.40	*b* = 0.062 ± 0.007	—	*a* = 1.46 ± 0.13	*b* = 0.062 ± 0.003	—	*a* = 2.18 ± 0.35	*b* = 0.035 ± 0.005	—
${\hat{JND}}_{\mathrm{ME}}$	*a* = 4.24 ± 0.54	*k* = 0.023 ± 0.014	—	*a* = 4.72 ± 0.58	*k* = 0.035 ± 0.011	—	*a* = 3.35 ± 0.12	*k* = 0.027 ± 0.003	—	*a* = 3.72 ± 0.83	*k* = 0.062 ± 0.010	—
ADJ	—	—	—	—	—	—	—	—	—	*a* = 45.34 ± 0.42	*b* = 1.07 ± 0.02	*c* = −0.0003 ± 0.0003
*SD*_ADJ_			—	—	—	—	—	—	—	*a* = 0.64 ± 0.23	*b* = 0.054 ± 0.01	—
${\hat{JND}}_{\mathrm{ADJ}}$			—	—	—	—	—	—	—	*a* = 0.50 ± 0.29	*k* = 0.055 ± 0.013	—

## Appendix D. Number of participants and trials for each task in each dataset

**Table D1.** Participants and trials for each task in each dataset.

Task	Experiment 1	Löwenkamp et al. 2015	Heath et al. 2011	Ganel et al. 2008
Grasping				
Participants	20	15	16	13
Trials	50	20	20	20
Manual estimation				
Participants	20	15	11	11
Trials	50	20	20	20
Perceptual adjustment				
Participants				6
Trials				20

## Appendix E. Fitting strict versions of Weber's law

### Linear fit with zero-intercept

As described in the main text, the question might arise whether it would be better to fit Weber's law with a strictly zero-intercept, such that

$$\begin{array}{c} \hat{JND}\left(s\right)=ks \end{array}$$

(Compare to Equation 12 of Appendix A to see the difference to the standard method based on the generalized version of Weber's law.) Although this zero-intercept method would be unusual (Brown et al., 1962; Miller, 1947), we implemented it for the current data (Table E1). Inspection shows that the corresponding Weber constants are much larger than those obtained by the standard method (compare Table E1 to Figure 4 in the main text and Appendix C). The standard method used by us therefore provides rather conservative estimates of the Weber constants.

**Table E1.** Results of Weber constants *k* (mean ± *SEM*) with zero-intercept regression between $\hat{JND}$ and object size.

Task	Experiment 1	Löwenkamp et al. (2015)	Heath et al. (2011)	Ganel et al. (2008)
Grasping	0.138 ± 0.007	0.152 ± 0.009	0.175 ± 0.174	0.097 ± 0.006
Manual estimation	0.132 ± 0.007	0.127 ± 0.007	0.101 ± 0.013	0.133 ± 0.017
Adjustment	—	—	—	0.064 ± 0.01

**Table E2.** Weber constants *k* (mean ± *SEM*) obtained from the alternative method based on Smeets and Brenner (2008).

Task	Experiment 1	Löwenkamp et al. (2015)	Ganel et al. (2008)
Grasping	0.069 ± 0.011	0.085 ± 0.014	0.039 ± 0.010
Manual estimation	0.055 ± 0.011	0.062 ± 0.011	0.090 ± 0.012
Adjustment	—	—	0.058 ± 0.010

### Alternative method of Smeets & Brenner (2008)

During the review of this manuscript, one reviewer (Jeroen Smeets, signed review) suggested an interesting alternative analysis that was initially proposed by Smeets and Brenner (2008). In a nutshell, they suggested modeling the $\hat{JND}$ as a combination of two independent variances, one constant (intercept term) and one depending on stimulus intensity. This gives a nonlinear relationship between $\hat{JND}$ and stimulus size:

$$\hat{JND}\left(s\right)=\sqrt{a^{2}+\;k^{2}s^{2}}$$

with *a* being the constant source of variability and *k* being interpreted as Weber's constant. The equation can be reformulated to

$$\hat{JND}{\left(s\right)}^{2}=a^{2}+k^{2}s^{2}$$

such that a simple linear regression can be performed on these quadratic terms, with *a*^2^ corresponding to the intercept and *k*^2^ corresponding to the slope (for further details, see Smeets & Brenner, 2008). A small glitch can occur when the regression results in negative values for *a*^2^ and *k*^2^, such that *a* and *k* are undefined. In these cases, reduced models with the corresponding parameter set to zero were fitted (e.g., if *a*^2^ would be negative in the full model, then a model with zero intercept is fitted).

The reviewer suggested using the Smeets and Brenner (2008) method instead of the standard method as has been employed in our study (cf. Equation 12 of Appendix A), as well as in most psychophysical studies (Brown et al., 1962; Miller, 1947). We therefore implemented this method for those studies where full per-participant data were available (Table E2). Results are comparable to the results obtained by the standard method (compare Table E2 to Figure 4 in the main text and Appendix C), but consistently larger. The standard method used by us therefore provides rather conservative estimates of the Weber constants.

### References

Brown, R., Galanter, E., Hess, E. H., & Mandler, G. (1962). New directions in psychology. New York: Holt, Rinehart, & Winston.

Ganel, T., Chajut, E., & Algom, D. (2008). Visual coding for action violates fundamental psychophysical principles. *Current Biology,*
*18*(14), R599–R601, https://doi.org/10.1016/j.cub.2008.04.052.

Heath, M., Mulla, A., Holmes, S. A., & Smuskowitz, L. R. (2011). The visual coding of grip aperture shows an early but not late adherence to Weber's law. *Neuroscience Letters,*
*490*(3), 200–204, https://doi.org/10.1016/j.neulet.2010.12.051.

Löwenkamp, C., Gärtner, W., Haus, I. D., & Franz, V. H. (2015). Semantic grasping escapes Weber's law. *Neuropsychologia,*
*70*, 235–245, https://doi.org/10.1016/j.neuropsychologia.2015.02.037.

Miller, G. A. (1947). Sensitivity to changes in the intensity of white noise and its relation to masking and loudness. *The Journal of the Acoustical Society of America,*
*19*(4), 609–619, https://doi.org/10.1121/1.1916528.

Smeets, J. B. J., & Brenner, E. (2008). Grasping Weber's law. *Current Biology,*
*18*(23), R1089–R1090, https://doi.org/10.1016/j.cub.2008.10.008.
